# Supplementary material for: Circulating miRNome detection analysis reveals 537 miRNAS in plasma, 625 in extracellular vesicles and a discriminant plasma signature of 19 miRNAs in children with retinoblastoma from which 14 are also detected in corresponding primary tumors
Source: PLoS One. 2020 Apr 14;15(4):e0231394. doi: 10.1371/journal.pone.0231394 (PMC7156076; doi:10.1371/journal.pone.0231394)
Supplement: Attachment — Submitted filename: Response to reviewers.docx [file pone.0231394.s007.docx]

Mexico City February 25, 2020

Dear Editor and Reviewers,

We wish to thank the reviewers for their careful and thoughtful critiques. Below you will find our point by point response to their critiques, the detailed responses in blue to reviewers and highlighted in yellow the location in the manuscript of the modifications. During the analysis requested by reviewer #2, we realized there was a mistake in the sex of one dataset, we corrected it and change accordingly our findings: in figure the number of sex related miRNAs in females from 7 to 5 as well as in the abstract. We also notice one reference was missing, and we corrected it too. We feel that the revised manuscript has been strengthened through our response to the comments.

We hope that the editorial board will agree on the interest and scope of this study and find the work acceptable for publication at the PlosOne journal.

On behalf of all the authors

Verónica Ponce-Castañeda M.D., Ph.D.

**Reviewer #1:** Castro-Magdonel and colleagues carried out a survey on miRNAs from retinoblastoma children’s plasma or extracellular vesicles and compared the miRNAs patterns with healthily age matched children, which provided us an informative resource of the landscape analysis of miRNAs in retinoblastoma children. However, there are some concerns should be addressed before consideration for publication.

The title of the manuscript is too long. Modifications required.

Response= We modified the manuscript title from a 30 word (233 characters) to 22 words (137 characters):

**Plasma miRNome analysis from children with retinoblastoma reveals the size of the circulating miRNome, a tumor signature and novel candidates for normalizers.**

Line 35: Circulating miRNAs are potential useful biomarkers in medicine.

Why they are only important in medicine? The authors should include all the related fields miRNAs involved.

R= we agree that miRNAs are important in many fields beyond medicine, to indicate this we included the following in the abstract’s opening statement in the abstract:

“miRNAs regulate post-transcriptional gene expression in metazoans, and thus are involved in many fundamental cellular biological processes. Extracellular miRNAs are also found in most human biofluids including plasma. These circulating miRNAs constitute a long distance inter cellular communication system and are potentially useful biomarkers.”

Line 39: To address these questions,

It seemed that the authors did not mention any questions or issues regarding miRNAs in previous couples’ sentences. Clarify them.

R= we rearranged the text as follows:

We proposed to answer how many and which miRNAs are detectable in plasma or extracellular vesicles as these questions have not yet been answered. We set out to address this knowledge gap by analyzing the mirRNome in plasma and corresponding extracellular vesicles (EVs) from 12 children….

Line 67: In plasma have been found both, in soluble form associated to Argonaut proteins, and also inside extracellular vesicles or exosomes (6)(7). This sentence is hard and difficult to understand. Make it readable.

*R= we agree and have modified the sentence as follows:*

“In plasma, miRNAs associate with Argonaut proteins and can also be detected inside extracellular vesicles or exosomes (6)(7).”

Line 88 to Line 103:

The authors attempted to make their methodology more convincing, logic, and fit their study very well, but they failed. Recommends revision and rearrangements to make it concise and precise.

R= we understand the reviewer’s suggestion and have modified and rearranged the paragraphs as follows:

“High throughput expression microarray technology can scan complete miRNnomes or transcriptomes in a single hybridization experiment. The original technology used a double channel platform and yielded relative measurements. However, more mature technology with one channel platforms include calibrators which yield absolute measurements. These calibrators allowed the development of robust methods for detection calls (19) which are basically ignored by most analysis. Broad maps of what is and is not detected can now be generated with the detection score using clustering tools thus facilitating data interpretation (20). This approach allows answering relevant questions such as ‘how many’ and ‘which’ miRNAs can be detected in plasma or EVs? Similarly, one can now posit: ‘Are detectable miRNAs shared by all samples, phenotypes or experimental conditions?’

Materials and methods

The controls and the Rb patients’ age and sex are not matched well. Also, the body weight or BMI, and race should be included if it is not unavailable to the authors.

R= as noted in table 1, age and gender did not differ significantly between the 12 cases and the 12 healthy controls. Weight at time of blood draw was not available for all cases and controls. All children were of Hispanic ethnicity. We do not collect information on ‘race’ in the underlying study as we do not feel that it would be meaningful or easily interpretable in the context of our population which is expected to have high and variable racial admixture.

Further, in microarray experiments, variations of expression measurements can be attributed to many different sources that influence the stability and reproducibility of microarray platforms. How the authors handle this issue?

R= One of the motivations to do this study is the remarkable stability of miRNAs in plasma. The samples have been collected under standard procedures and kept at 4°C until plasma separation (within 6 hours of sample collection) and then kept frozen at -70 °C until RNA isolation. Our analytic approach focused first on generating the detection score that simplifies handling microarray data, and later on examination of expression measurements. In our pilot work, we performed technical replicas, and since both expression and detection data were highly correlated, we opted to prioritize more biological replicas in our design instead of additional technical replicas. Expression measurements in microarray platforms are very sensitive and detect large amounts of ‘noise’. We believe that this sensitivity masks underlying data patterns which are unmasked by using the detection score.

Is normalization applied when process the data?

R= Yes, we indicated this in the Data Analysis section Lines 147-148.

Results and discussions

There are several studies reported about 1000 to 2000 miRNAs in Rb patients’ plasma (<https://www.ncbi.nlm.nih.gov/> pmc/articles/PMC5493862/). Why the authors only detected about 600 miRNAs in plasma?

R=to our knowledge this is the first work reporting the plasma miRNnome from children with retinoblastoma. The article mentioned by the reviewer is our own previous report of the primary tumors which correspond to the samples mentioned in this manuscript. We have cited this article in our references as reference ‘(20)’. We used the same platform as described in that publication: Affymetrix chips miRNA 4 with 2578 miRNAs.

We do not know why do we are able to detect approximately 600 miRNAs in plasma. We can only speculate the reasons. Plasma can be considered a ‘liquid’ tissue, and as such only a subset of genetic elements from the total miRNnome are “expressed” or detected. It may follow the same tenet as that of tissue specificity, which is result from the expression of only a subset of the genes in the genome.

We realize that some information about the number of miRNAs per cluster shown in the detection map generated from the primary tumors is missing in Table 2. We have now added the corresponding data from the primary tumors.

Rb tissues	995	1441	142

How the authors validate the miRNA expression profile? Any qRT-PCR validation applied?

R= we used stem-loops and exiqon miRNA probes for 6 miRNAs with high expression levels from the 19 miRNA signature for RB cases. We obtained mixed results and only 3 miRNAs showed high detection levels with both experimental approaches. We think that qRT-PCR is not optimal for miRNA detection and believe that more reliable methods need to be developed.

The figures’ resolution is always low, which are difficult to read.

R=we apologize for the difficulty in reading our images. With this revised submission, we submit better images with higher resolution.

**Reviewer #2**: In this paper, the authors try to answer the questions what the differences are between the healthy person and Rb (diseased) person in terms of their miRNAs in plasma and extracellular vesicles using 12 patients and 12 controls. The size of miRNAs in plasma are 537 and in EV is 626. And they found that 19 miRNAs in plasma are significant between healthy and diseased person. And they also found 9 miRNAs that can be detected in all the samples, suggesting that these 9 miRNAs can be used as internal control. They also found that the gender is also a factor that affects the miRNAs between healthy and diseased person.

First, I think the English in this paper needs to be improved.

R= we apologize for the grammatical errors in our initial submission. We have carefully edited this revised submission and hope that it will be easier to read.

Second, I think using 12 patients and 12 control as samples are too few to get conclusion.

R= we recognize the sample size is small, however we believe our findings represent an important contribution that can advance the scientific field because we show a different analytic approach that opens up new possibilities for interpretation and permits extracting information that may potentially be useful in the field of using miRNAs as circulating biomarkers. We therefore consider our conclusions are informative and valid.

Third, the difference between the plasmid and EV in terms of its chemical components should be discussed in the paper.

R=the reviewer may refer to plasma instead of ´plasmid‘, we have now included the following text in the discussion:

“Our finding that miRNA contents in total plasma and EVs are distinct, may be particularly informative. One possible explanation may be that the purification of EVs may amplify signals from miRNAs that may not be detectable in miRNAs extracted from whole plasma. Importantly, the 19 miRNA signature that is able to discriminate Rb cases from healthy controls is found only in RNA from total plasma and not in EVs, this is relevant because the method to obtain RNA from plasma is simpler and less expensive than the method to obtain RNA from of EVs.”

Other than that, I have some comments as below:
1. Methods/Patients and samples: What are including criteria and what are excluding criteria?

R= we have now included the following text in the Methods, patients and samples section:

“Inclusion criteria: children newly diagnosed with retinoblastoma at an age of less than or equal to 6 years, without a known genetic syndrome or prior diagnosis of cancer, who were diagnosed or were receiving treatment at the two participating treatment centers, and did not have a preexisting/ known family history of retinoblastoma. One child later found to have familial retinoblastoma was included in the study.

Exclusion criteria: age greater than 6 years of age at time of diagnosis, known diagnosis of a genetic syndrome or prior diagnosis of cancer.”

2. How are the healthy control being selected?

R= Control selection used ‘friend’ controls as a proxy for population controls. Controls were children of women who were friends (but not a biological relative) of the mother of an eligible case child. Exclusion criteria for controls included being diagnosed with a known genetic syndrome, or having a family history of retinoblastoma, or a personal history of any type of cancer. Controls were within 2 years of age of the case child. We have included an abbreviated description of the source of the population at the beginning of the Materials and Methods section as we acknowledge that this should be included.

3. How many times the experiments for Fig 1, 2, and 3 are done? If they are only done once, how do the authors make sure the results are reliable?

R= in our pilot work, we performed a technical replica for 1 tumor (2 microchips) and for 1 plasma (2 microchips), and since expression and detection data had a very high correlation, we decided to include in the design more biological replicas to obtain more information on reproducibility and biological variability. We performed tumor experiments 12 times, plasma experiments 24 times and EVs experiments 24 times, we believe these make our results reliable.

4. In figure 3 and 4, how top is the top ranked miRNAs?

R=Figure 3 is a detection map generated with hierarchical clustering using the detection score for miRNAs isolated from EVs, there are no top ranked miRNAs in Fig 3.

Figure 4 shows heatmaps and volcano plots generated with the results of differential analysis using expression data. We used the expression ’top ranked’ to identify in the output results table, all significant over expressed miRNAs in the Rb cases. The results of Differential Analysis are lists of miRNAs with log2 transformed values, this transformation is performed to obtain symmetrical data, positive numbers for Rb cases, and negative numbers for healthy controls. We ordered this transformed values from large to small to identify at the top of positive values, those miRNAs over expressed in Rb cases.

To avoid confusion, we added the following:

In the differential analysis we found 158 differentially expressed miRNAs in plasma and 142 differentially expressed miRNAs in EVs. However, adjusted *p values* indicate that differences were not significant for any of them.

We also change the expression top ranked to ‘The most differentially expressed’ miRNAs in the table 3 and 4 titles.

We also added before description of figure 4 the following:

The corresponding heatmap does not show color differences among cases and controls except for the 158 and 142 differentially expressed miRNAs which are shown in **Fig 4.** Even though differential analysis identified miRNAs with p-values <0.05, the adjusted p-values did not show any statistically significant differences between cases and controls for any miRNA.

Are the top miRNAs showed in Table 3 and Table 4 same as those detected by mirnomic analysis?

R= Yes, the top miRNAs showed in Table 3 and Table 4 are the same as those indicated in the volcano plots in figure 4 b and d. We are submitting a better quality figure with larger fonts to enhance the readability of the miRNA identifiers.

5. In Figure 5 and table 5, the authors summarized the number of detected miRNAs per sample, per group (cases or controls) and per class. How does these numbers compare to other mirnome studies?

R=to our knowledge this is the first work reporting the size of plasma miRNome using the terms ‘detected’ miRNAs. We added in table 2 miRNome data from the corresponding primary tumors:

Rb tissues	995	1441	142

6. Figure 7 indicated that gender may be a factor that cause the miRNA to be different. So Could the authors analyze the difference of miRNA in plasma and EV between diseased person and health person with gender differences? So there will be four groups: 1) health female, 2) health male, 3) diseased female, and 4) diseased male.

R= We think that in order to interpret this findings, more balanced groups are needed with equal number of males and females in each group. Since our sex related findings were unexpected we didn’t consider this balance in the study design. Nonetheless we now submit the requested analysis and groups obtained with hierarchical clustering of detection scores and a summary of this analysis in the following table:

Group	Condition	No. of shared mirnas

Group1	Plasma healthy females	21

EVs healthy females	33

Group 2	Plasma healthy males	22

EVs healthy males	62

Group 3	Plasma Rb females	63

EVs Rb females	73

Group 4	Plasma Rb males	36

EVs Rb males	44

7. The authors found that 19 miRNAs shared exclusively by all Rb cases. Are these 19 miRNAs have any similarities? Which pathways did these 19 miRNAs targeted? How about this result compared with other similar results? Is there any difference? If yes, why?

R= from the 19 miRNAs in plasma that were shared by all Rb cases, 14 are also part of the 142 miRNA core shared by all the primary tumors described in (20). For the 19 miRNAs found, using mirPath v.3 we found 51 KEGG pathways with a wide variety of biologic functions which probably reflect the physiologic processes in which the circulating miRNAs are involved. There are no similar results in the literature.

8. Why “miR-16-5p signals are detectable in 17 / 21 samples mainly in EVs” but not all the samples in EVs? And why “miR-21 is detected only in a few samples” but not all the samples? Since miR-16 and miR-21 are all normalizers, they should be seen in all the samples?

R= miR-16 and miR-21 are the most common miRNA used as normalizers for plasma with qRT-PCR. Ideally a normalizer should be detectable in all the samples. Our data show that these two common normalizers are not detected in all samples. However, we found 9 miRNAs that were detectable in all the samples, making these nine better candidates to be used as normalizers. We do not know why miR-16 and miR-21 are not detectable in all of our samples.

9. Line 319: “13 miRNAs as a signature are exclusively detected in EVs from Rb cases.” Please delete exclusively because it is not exclusive as 12miRNAs are also seen in healthy controls.

R= we agree that the word produces confusion, our apology for this we eliminated it.

10. Why “more robust circulating Rb case signature is in the plasma compartment not in EVs”? Could the authors give an explanation?

R= this work essentially describes what can be detected in plasma and EVs using the detection score. At this moment we can only speculate about why the plasma signature clearly better discriminates Rb cases from healthy controls compared with the EVs signature. One possibility is that detectable miRNAs in plasma might reflect both the circulating tumor message as well as a systemic response to cancer.

11. Line 302-303: “the differences are found not too strong” is too subjective. Please revise this sentence, e.g., adding a reference.

R= We change the sentence as follows:

“The corresponding heatmap does not show color differences among cases and controls except for the 158 and 142 differentially expressed miRNAs which are shown in **Fig 4.** Even though differential analysis identified miRNAs with p-values <0.05, the adjusted p-values did not show any statistically significant differences between Rb cases and controls for any miRNA.”

12. Table 1: Did the authors do the statistical analysis on the patient gender between the health and diseased group?

R= we compared the sex distribution between cases and controls and found no statistical difference. We have now added this in the footnote to the table.

13. EVs includes exosomes and microvesicles. In the introduction, the authors only introduce the exosomes but not the microvesicles. Please add some information of microvesicles.

 R= we arranged information in the introduction stating that:

“In plasma, exosomes are part of extracellular vesicles (EVs) that include particles of diverse sizes and origins which are not fully characterized. There is not a standard definition of what exosomes are. In practical terms these are mixed populations of membranous nanosized particles about 50-130 nm in diameter (8)(9)(10).
